# Creating a Medical Imaging Workflow Based on FHIR, DICOMweb, and SVG

**DOI:** 10.1007/s10278-021-00522-6

**Published:** 2023-02-02

**Authors:** Shih-Tsang Tang, Victoria Tjia, Thalia Noga, Jeshika Febri, Chung-Yueh Lien, Woei-Chyn Chu, Chin-Yu Chen, Chia-Hung Hsiao

**Affiliations:** 1 Department of Biomedical Engineering, Ming-Chuan University, Taoyuan, Taiwan; 2 Department of Medical Informatics, Tzu Chi University, 701 Zhongyang Rd. Sec. 3, Hualien, 97004 Taiwan; 3 Department of Information Management, National Taipei University of Nursing and Health Sciences, Taipei, Taiwan; 4 Department of Biomedical Engineering, National Yang Ming Chiao Tung University, Taipei, Taiwan; 5 Department of Radiology, Chi-Mei Medical Center, Tainan, Taiwan

**Keywords:** FHIR, DICOMweb, RESTful, Scalable Vector Graphics (SVG)

## Abstract

This paper proposes a web-based workflow scheme for the organization of medical images using FHIR and DICOM servers equipped with standard RESTful APIs. In our integrated workflow, the client systems (including order placer, scheduler, imaging modality, viewer, and report creator) use standard FHIR and DICOMweb APIs. The proposed scheme also facilitates the creation of reports formatted as standard FHIR resources. This paper leverages W3C Scalable Vector Graphics (SVG) to record the image graphic annotations, and encapsulates the SVG image annotation in FHIR observation. FHIR DiagnosticReports and Observations are used to encapsulate reports, findings, and annotations, thereby facilitating the implementation and integration of the scheme within existing structures. The proposed scheme also provides the potential to make it possible to convert results of Computer Aided Detection/Diagnosis from medical images into FHIR DiagnosticReports and Observations to be stored on a FHIR server. The resulting web-based solution uses FHIR XML and/or JSON data to record and exchange information related to imaging workflow. It can also be used to store imaging reports, findings, and annotations linked to the images using the DICOM WADO-RS protocol. As a result, it is possible to integrate all information that is created in medical imaging workflow. Finally, the proposed scheme is easily integrated with other FHIR systems.

## Purpose

In Integrating the Healthcare Enterprise (IHE) Scheduled Workflow (SWF) [1], medical imaging workflows must follow HL7 V2 (Health Level 7 Version 2.x) and DICOM (Digital Imaging and Communications in Medicine) protocols for the sharing of data. The SWF profile addresses actors (information systems) that support HL7 V2 and DICOM protocols for workflow and medical image management. The XDS or XDS-I profile (IHE Cross-Enterprise Document Sharing) is also used for the sharing of medical images outside the hospital [1, 2]. The previous versions of HL7 and DICOM standards are very large and complex. It’s not easy to implement the imaging workflow handling and imaging data sharing. Thankfully, DICOM and HL7 have issued web RESTful (REpresentational State Transfer) specifications and proposals, which are respectively referred to as DICOMweb (DICOM Web Services) and FHIR (Fast Healthcare Interoperability Resources) [3, 4]. These efforts have made it friendlier to develop an integrated imaging workflow.

In this study, we developed a demonstration system in which FHIR and DICOMweb specifications are used to facilitate the integration of conventional HIS (Hospital Information System) and PACS (Picture Archiving and Communication System). Our workflow scheme proposes that the HIS implements server-side support for several FHIR resources and the PACS implements server-side support for several DICOMweb services. The storage and handling of imaging workflow and report data is performed on the FHIR server, whereas medical images are stored on the DICOMweb server for distribution. Thus, it is possible to use standard FHIR XML (eXtensible Markup Language) or JSON-formatted (JavaScript Object Notation) data to record information created during imaging workflows. Additionally, we employed FHIR and DICOMweb RESTful APIs (Application Programming Interface) to facilitate integration with existing systems. The proposed web-based solution is easily implemented, and the DICOMweb-based image sharing is easily integrated with other workflows pertaining to clinical diagnosis and treatment.

## Methods

### Conventional Imaging Workflows

**Fig. 1:**
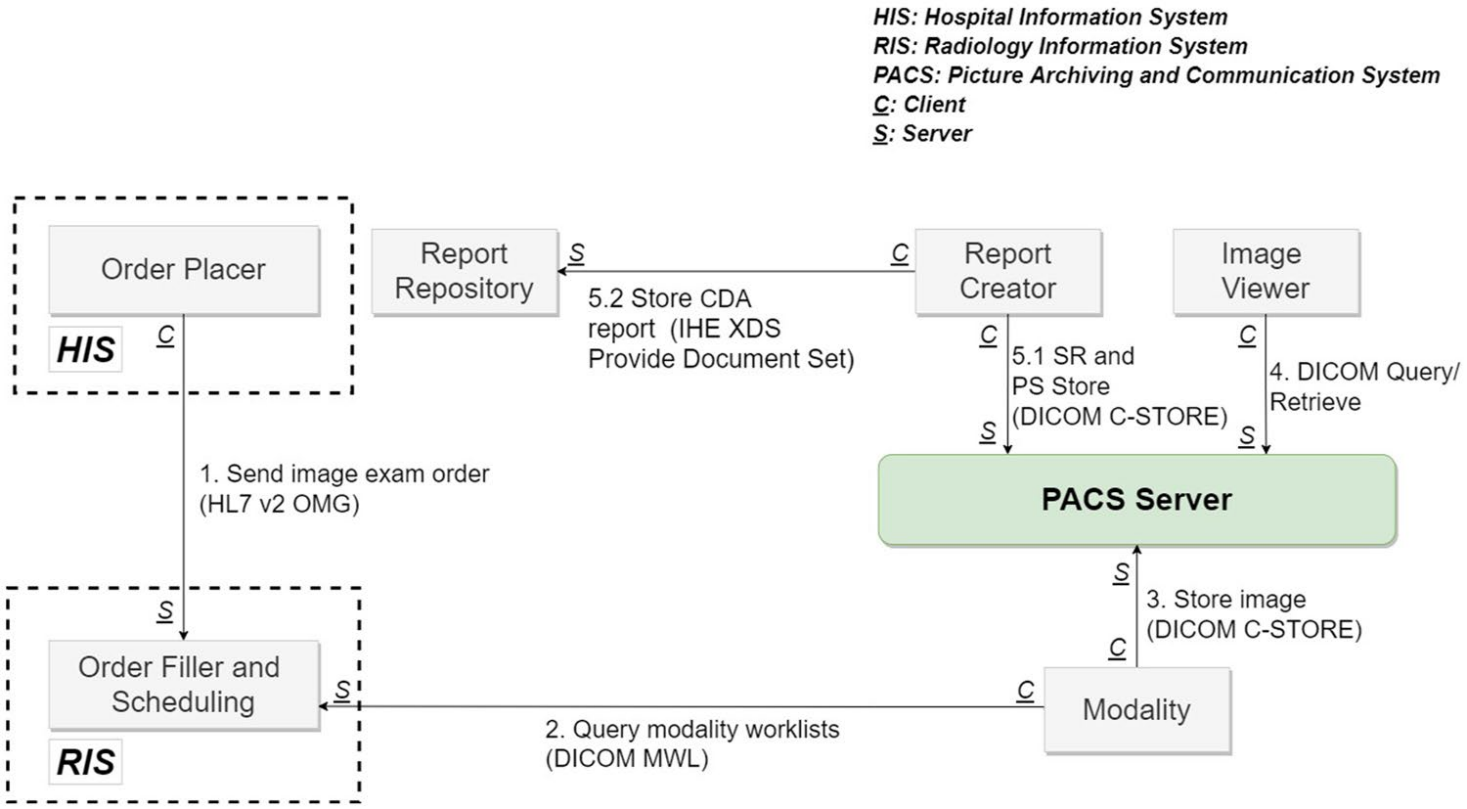
Conventional medical imaging workflow

Conventional medical imaging workflows require the integration of information systems. As shown in Fig. 1, standardized data is created in the HIS, RIS (Radiological Information System), and PACS, and then shared across systems based on IHE Integration Profiles.

Figure 1 lists the six fundamental systems that must be integrated within a typical imaging workflow. The six systems deal with ordering, scheduling, imaging, storing, inspecting, and reporting. In accordance with the IHE Radiology SWF and Reporting Workflow (RWF) profiles [5], system integration requires support for the DICOM protocol (modality work list, image storage, image query/retrieve) as well as the HL7 V2 protocol (ordering and image availability notification). The system handles imaging reports usually via PDF file, the HL7 Clinical Document Architecture (CDA), or DICOM Structure Reporting (SR) for the storage of reports in the report repository or PACS. It is also important to provide support for IHE XDS or XDS-I profiles using ebXML (Electronic Business using eXtensible Markup Language) web services for the sharing of images and reports among hospitals.

### FHIR and DICOMweb Imaging Workflows

**Fig. 2:**
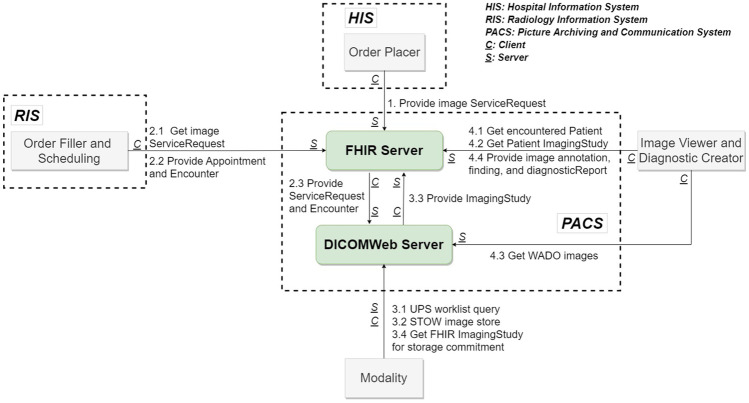
Proposed medical imaging workflow

In this study, we developed a novel web integration framework based on recent medical informatics standards, HL7 FHIR and DICOMweb. As shown in Fig. 2, the proposed framework allows straightforward implementation, management, and integration with existing systems used in the healthcare environment.

The proposed framework includes two web servers (FHIR and DICOMweb servers) and four clients (Order Placer, Order Filler, Modality, and Viewer). All of the clients and servers use the HL7 FHIR and DICOMweb protocols to facilitate integration, and both servers utilize HTTP RESTful APIs for the sharing of data, which is formatted as XML or JSON and stored on the FHIR server. Data sharing by the DICOMweb server can be in XML, JSON format, or binary data format. Our adoption of RESTful web solutions makes it easy for most information technology (IT) developers to understand the system. In accordance with HL7 FHIR, DICOMweb, and IHE, the data creation and exchange processes are as follows:

Step 1.  The Order Placer (HIS) initiates an order for imaging by creating a corresponding FHIR ServiceRequest on the FHIR Server (HIS).

Step 2.  The order is handled by the Order Filler (RIS) as follows:

Step 2.1  The Order Filler periodically queries the FHIR Server for new ServiceRequests

Step 2.2  RIS schedules an imaging appointment and creates a new EncounterResource on the FHIR server.

Step 2.3  The FHIR server maps the information in the ServiceRequest and Encounter Resources into a client request to the DICOMweb Server to create the UPS-RS worklist item. [6]

Step 3.  Modality imaging procedure:

Step 3.1  Modality obtains a UPS-RS work list from the DICOMweb server.

Step 3.2  Following completion of the imaging session, the images are stored in the DICOMweb Server via STOW (STore Over the Web). [7]

Step 3.3  Mapping from a DICOM Study to the FHIR ImagingStudy Resource.

Step 3.4  Finally, modality uses QIDO-RS to ensure that the images are stored on the server. Note that this is similar to the DICOM storage commitment used in PACS [8].

Step 4.  Image retrieval and reporting

As shown in Fig. 2, radiologists can use the image viewing and reporting system to obtain images and create reports. This process is described in the following:

Step 4.1  The radiology department queries the FHIR encounter resources to prepare the prioritized worklist for radiologists.

Step 4.2  Radiologist examines the images, and may get additional FHIR ImagingStudies (current and previous image exam results) from FHIR server, which might be pre-fetched.

Step 4.3  The viewing system retrieves the selected images via WADO-RS basing on QIDO-RS. This allows the radiologist to browse all of the medical images available for a given patient. [9]

Step 4.4  While the radiologist is inspecting the images, they may make annotations on medical images, describe the findings based on the annotations, and create a report of the image exam. According to FHIR standard, each image finding can be represented as a FHIR observation resource. And FHIR DiagnosticReport may reference to all the findings of the image exam. This paper suggests that image annotations also be represented as FHIR observation resource that can be referenced by FHIR finding observations. Consequently, image annotations, image findings, and report are correlated to each other. Report is referenced to image findings, and image finding may be referenced to image annotations. The image annotation (FHIR observation), image finding (FHIR observation), and report (FHIR DiagnosticReport) all could be stored in FHIR Server.^1^

### FHIR Solution for Interoperability

**Fig. 3:**
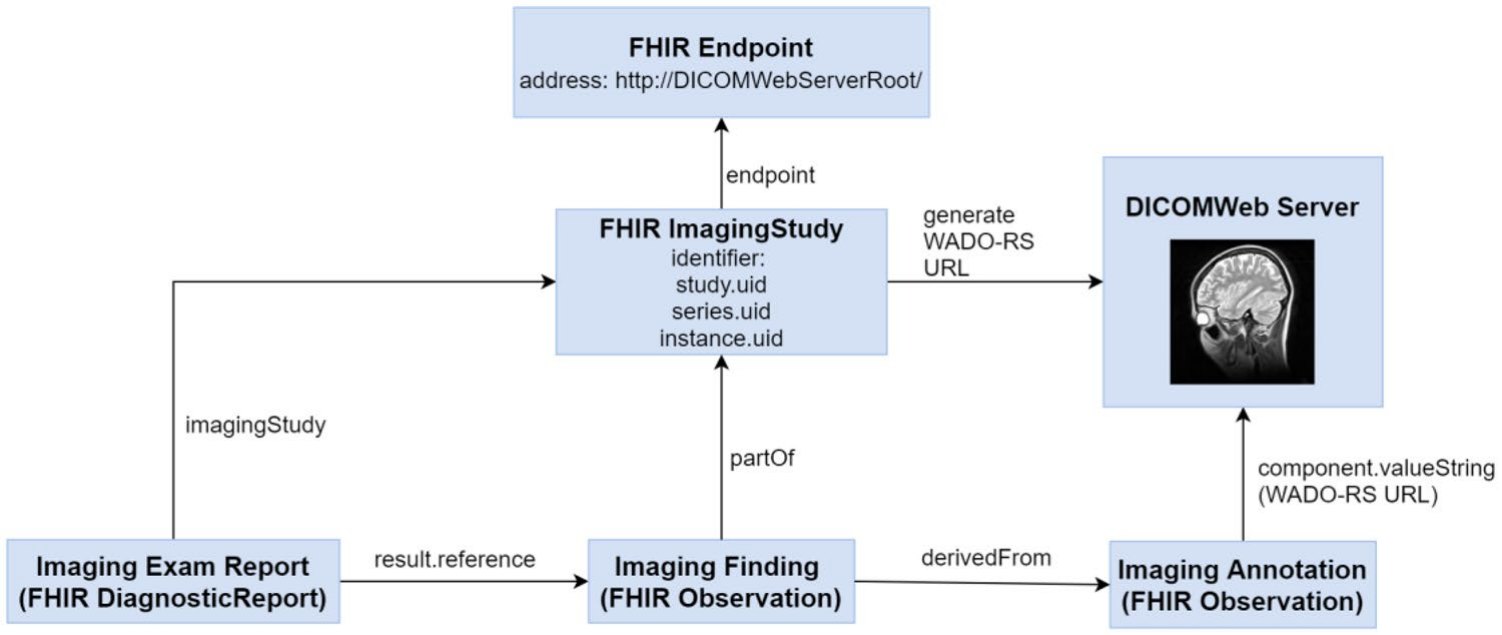
Proposed FHIR solution for linking annotations, findings, reports, and medical images

This paper outlines a novel FHIR solution aimed at facilitating the recording and archiving of data related to medical imaging. As shown in Fig. 3, the reports, findings, annotations, and DICOM images could be referenced together based on standards specified in the FHIR resources.

FHIR DiagnosticReport.ImagingStudy refers to ImageStudies associated with the report, DiagnosticReport.Result refers to findings (observations), and Observation.DerivedFrom (image findings) is derived from other observations containing annotation data pertaining to a specific medical image. The annotation Observation.Focus should reference to a WADO-RS URL (Uniform Resource Locator) that represents the focus image. We will show the SVG benefits in the following examples that package SVG graphics into FHIR observation for medical image annotation. This solution is easier for developers to understand and implement than DICOM GSPS for image annotation.

### SVG Annotation

**Fig. 4:**
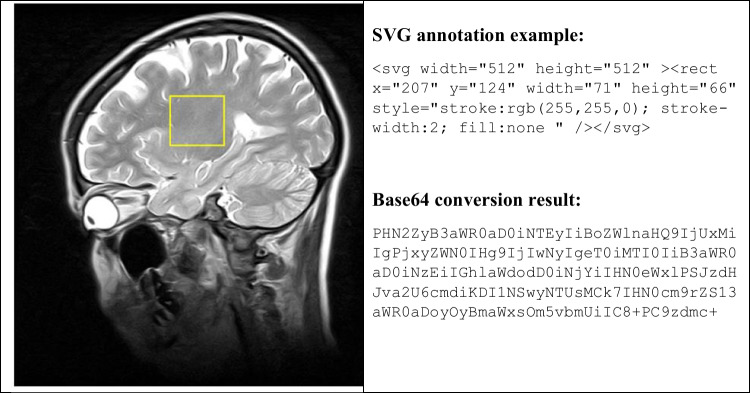
SVG annotations pertaining to a medical image

As described in Step 4.4 above, the radiologist can use the viewer to inspect medical images related to a particular patient and a report creator to make annotations pertaining to the images. The annotations are represented in Scalable Vector Graphics (SVG) format in accordance with W3C (World Wide Web Consortium) standards. Note that SVG annotations can be presented directly in a browser, and are easily dealt with by developers. Adding SVG data to observations in FHIR XML or JSON format requires that the SVG data be base64 encoded for storage in FHIR Observation, as shown in Fig. 4.

Figure 4 presents an SVG rectangle on an MRI (Magnetic Resonance Imaging) image. Note that such images are stored on a DICOMweb server and accessed in accordance with the DICOM WADO-RS protocol. A web-based viewer can use JavaScript to access DICOM images that are stored in DICOMWeb server. However, the annotation would not be stored into DICOMWeb server. According to DICOM standard the annotation should be DICOM Structure Reporting (SR) or Presentation State (PS) formatted for storing into DICOMWeb server. Unfortunately, DICOM objects are too complex to be directly handled by the general Web browser. And most system developers are unfamiliar with DICOM standards. Thus, we suggest storing image annotations in W3C SVG format, which feature clearly defined geometric graphics, such as lines, ellipses, rectangles, and polygons, and are familiar to most IT developers. This would also make it possible to create a simple SVG webpage with links to WADO-RS images containing geometric graphic annotations, as shown in the following script example:



Opening Example script 1 on a browser would render the image presented on the left side of Fig. 4. Note that in this example, < image x = "0" y = "0" width = "512" height = "512" xlink:href = "medicalImage.jpg" / > , where medicalImage.jpg refers to a background jpg image converted from a DICOM image object, and < rect x = "207" y = "124" width = "71" height = "66" style = "stroke:rgb(255,255,0); stroke-width:2; fill:none"/ > refers to the rectangle with a yellow border 2 pixels in width. For keeping the graphic annotation correctly viewed in different zooming situation. The graphic drawn on the viewer is transformed into original DICOM image coordinate before the SVG annotation uploaded to FHIR server. Note that the W3C SVG standard allows graphics that are supported by most existing browsers (e.g., Chrome or Firefox). However, the SVG annotation and WADO-RS image might also be presented in an application based viewer. The self-developed viewer might not work as a browser that supports all SVG specifications defined in W3C. Thus, a subset should be defined to interoperate the W3C SVG specifications and medical image annotation, which would be discussed in further investigation.

### FHIR Observation Using SVG Annotation

We suggest that SVG annotations are contained within FHIR Observation for storage on an FHIR server. The annotations usually can be stored as three methods: (1) SVG format in a FHIR Observation, e.g., retrieve annotations from a FHIR DiagnosticReport resource and displaying them directly. (2) SVG or DICOM-compatible formats as a reference stored in ImagingStudy.Endpoint in an ImagingStudy resource. (3) DICOM-compatible formats in the DICOMWeb server by referring to the URL of WADO-RS/WADO-URI. Under the (2) and the (3) methods, the annotations are retrieved via WADO, a DICOM to SVG broker to convert DICOM-based annotation into SVG coded as FHIR SVG observation, which need an additional server for storing the SVG annotation. As shown in Fig. 3, the annotation (FHIR Observation) can be referred by the image finding (another FHIR Observation), and the FHIR Observation.valueString could be used to store annotation information. Note that FHIR Observations should be in XML or JSON format, and W3C SVG graphics should be in XML format. Note also that containing SVG data directly to XML or JSON FHIR Observation.valueString would cause an error when uploading annotations to the FHIR server. Thus, we suggest that SVG data to be encoded in base64 to allow the storing of SVG data into FHIR Observations. The results could then be uploaded to the FHIR server, as illustrated in Example script 2 below.



Example script 2 is a simple FHIR image annotation encoded in base64 and stored in FHIR Observation.valueString. The valueBase64binary formatted data could be treated as a special type of string. Decoding the base64 string would restore the SVG annotation data to the form shown in Fig. 4. This FHIR Observation has been uploaded to an FHIR testing server, and can be accessed at the following URL: https://hapi.fhir.tw/fhir/Observation/4961

In Example script 2, Observation.Focus refers to a URL pointing to a DICOMweb server. The URL was created in accordance with the specifications for FHIR reference data, allowing it to be stored on an FHIR server. As shown in Fig. 3, the image annotation observation should be referred by finding observation and could be referred by FHIR DiagnosticReport.

When using a viewer to create FHIR annotations, the observations should contain only SVG data (i.e., without references to WADO-RS image), as shown in Fig. 4. The URL of the target image is stored in Observation.Focus. When deploying the image and annotation results outside the original FHIR and DICOMweb environment, we suggest using a standard front-end web solution compatible with standard browsers. Example script 1 presents a very simple solution showing one image and the corresponding annotation. This scheme can be deployed using HTML (Hyper Text Markup Language) and JavaScript to handle DICOM data objects. This would make it possible to create a portable viewer capable of showing DICOM images and annotations without having to deal with DICOMweb or FHIR servers.

## Results

### Viewer with Standard HTTP APIs

**Fig. 5:**
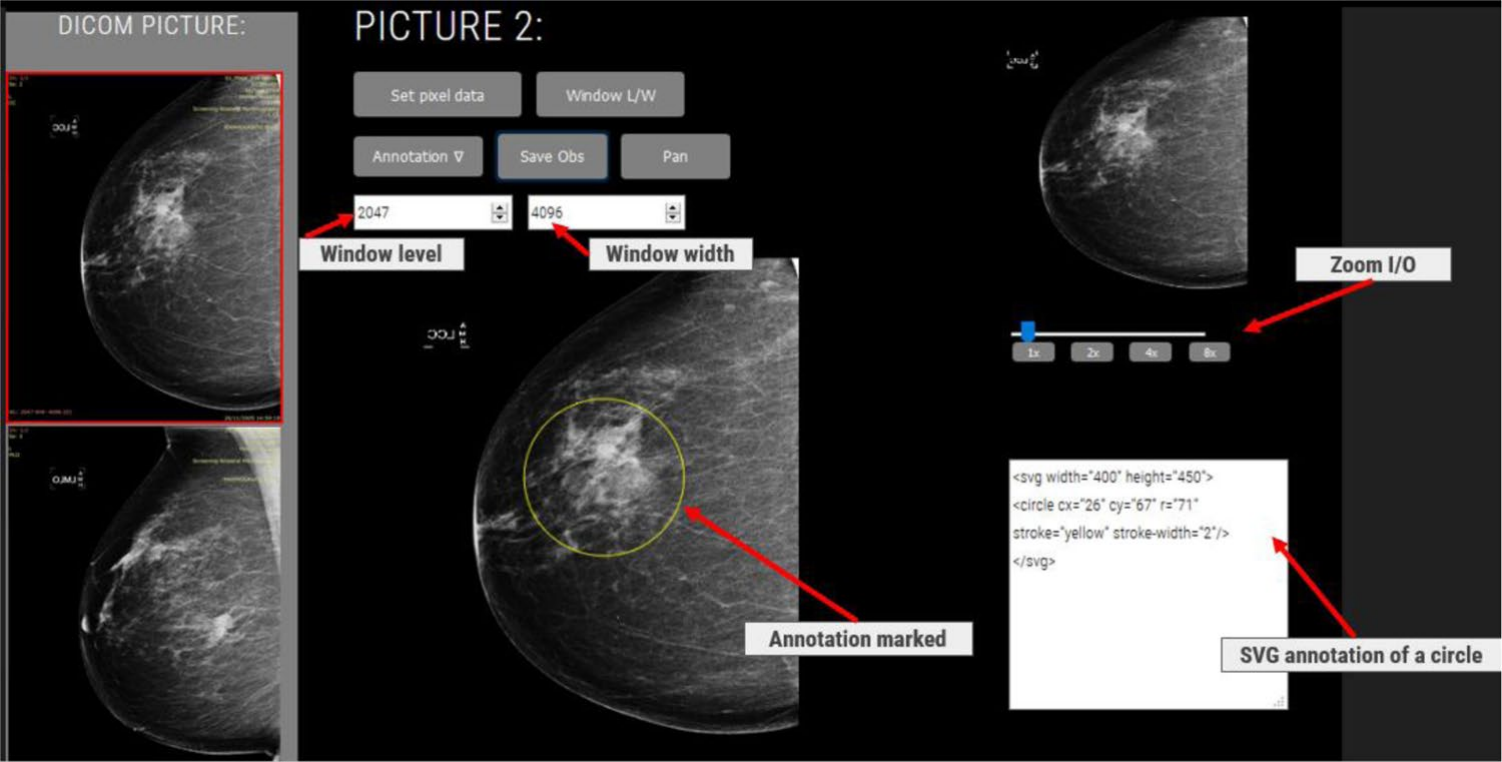
FHIR and WADO-RS viewer

As outlined in Step 4 above, we developed a viewer capable of querying and retrieving medical images as well as storing annotations and reports (see Fig. 5).

The above viewer provides functionalities of panning, zooming, window leveling, and annotating; however, FHIR APIs can also be used to obtain additional information pertaining to the patient (Step 4.1 above). After identify a patient for inspection, we could query all patient image studies use FHIR get image API (Step 4.2). Basing the query results, we can use WADO-RS to retrieve current and previous exam images (Step 4.3). Finally, the viewer can store annotations in the form of FHIR Observations on the FHIR server (Step 4.4). Conventional PACS viewers use DICOM query/retrieve protocols to access images and the DICOM store protocol to store annotations (DICOM PS objects) or reports (DICOM SR object), both of which are unfamiliar to most developers. The viewer presented in this paper uses FHIR specifications for querying and storing image reports on an FHIR server. Most FHIR developers are familiar with RESTful formatted APIs and XML or JSON formatted resources. Note also that the FHIR-based viewing and reporting systems could be easily integrated with other FHIR systems, such as medication, laboratory, and care planning systems.

### Converting DICOM SR into FHIR Resources

Computer-Aided Detection (CAD) systems can also be used in the acquisition and manipulation of diagnostic images. CAD systems may follow DICOM standard to convert the detection results into DICOM SR objects for storage on a PACS server. DICOM SR objects formulated under DICOM Part 16 specifications are very complex. Furthermore, CAD results should include data pertaining to the devices, procedures, measurements, subjects, and reference image information within a single DICOM SR object. This inevitably leads to the duplication of data stored in DICOM SR formatted CAD result. In this paper, we proposed a simplifying method that uses the FHIR resources, which would provide the potential to store CAD results on an FHIR server. As shown in Fig. 3, a FHIR imaging report contains FHIR DiagnosticReport, Observations (image finding and annotation), and related imaging studies. The CAD method and device information can be referred by FHIR Observations. Consequently, do not require to create the duplicate information when CAD results were created.

**Fig. 6:**
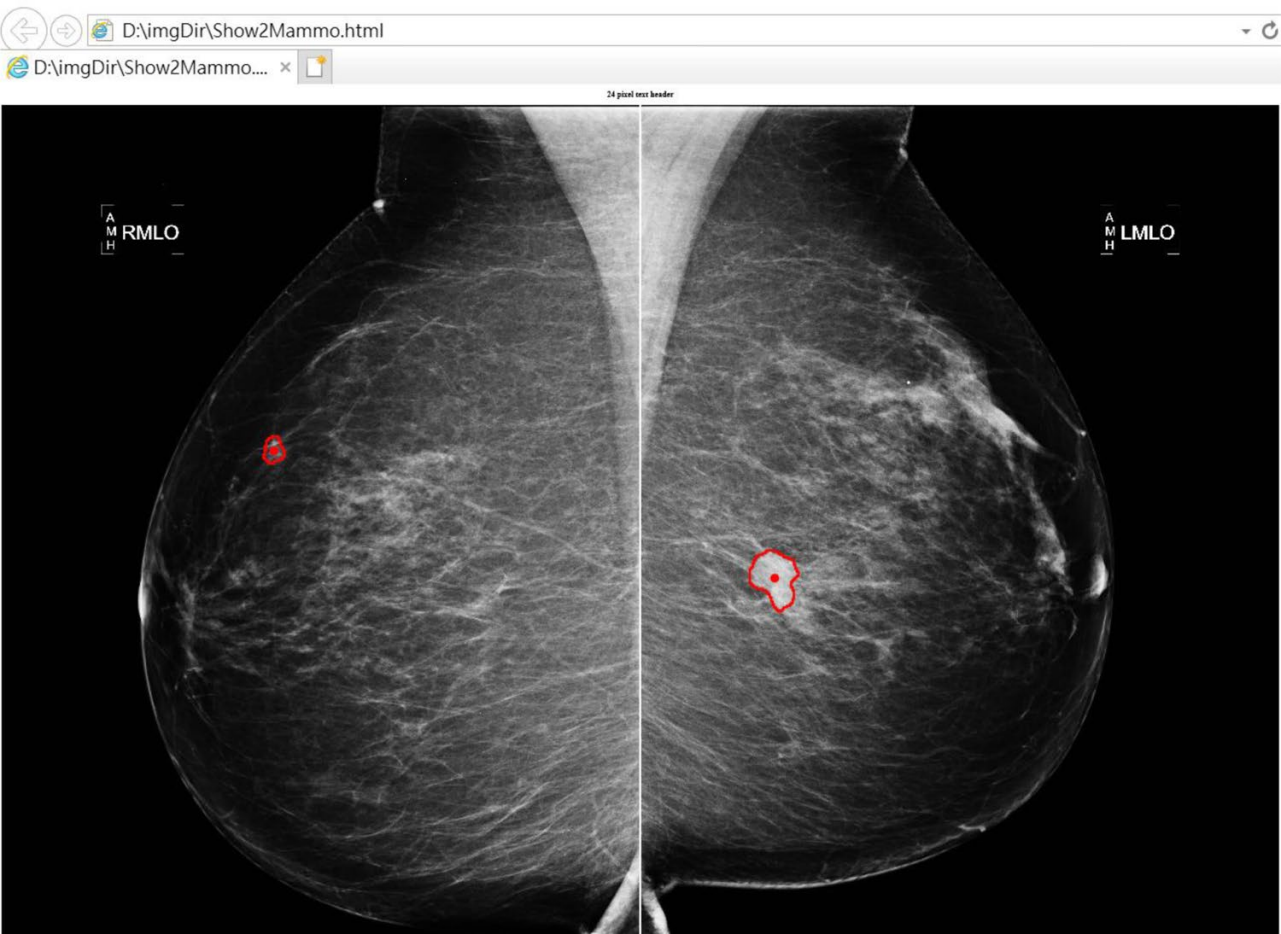
FHIR Observations and corresponding jpg mammographic images

Presenting CAD results in the form of FHIR Observations would be far simpler than using DICOM SR, as illustrated by the conversion of CDA DICOM SR data into FHIR Observations in Fig. 6. The illustration presents a HTML web page contains two FHIR annotations corresponding to two mammographic images. Note that the FHIR image annotation is easily integrated with FHIR DiagnosticReports as well as other (e.g., laboratory, pathology, and genomic) reports. In other words, the proposed scheme makes it possible to use a browser or client application to view any clinical results stored as standard FHIR DiagnosticReports and Observations. The current implementation simplifies by just rendering the contour. Future work would address migrating more of the omitted DICOM SR CAD complexity into FHIR Observations, e.g. CAD operating point, finding certainty, verified yes/no, algorithm name, presentation required/optional, size information, type of finding, summary of detections, etc.

As outlined in Fig. 3, CAD DICOM SR objects can also be converted into FHIR annotation Observations, finding Observations, and DiagnosticReports. Following the conversion, the CAD results are stored on an FHIR server. According the workflow in Fig. 2, the web solution described in this paper makes it possible for the viewing the CAD results and mammographic images on browser simultaneously. And as that shown in Fig. 6 and Example script 3, we can use pure web page to present CAD results and mammography on browser. The procedures involved in querying previous imaging results and presenting them in a pure web page are described in the following.

1. The viewer queries previous imaging DiagnosticReports for the patient of interest.
2. The viewer obtains the corresponding FHIR annotation Observations by the references in finding Observation and DiagnosticReport.
3. The viewer creates a WADO-RS URL using the ImagingStudy.Endpoint and annotation Observation.Focus, as shown in Example script 2.
4. The viewer retrieves the WADO-RS images for display.
5. The viewer draws annotations over the images.
6. The viewer exports a pure web page containing the target image and corresponding annotation.

Note that the above Procedure 6 (exporting a web page with images and SVG annotations) could be altered to allow the export of the original FHIR DiagnosticReports, Observations, and medical images in the form of web page with JSON data and JavaScript accessible using any browser. In other words, the results could be deployed to any website that does not support FHIR or WADO-RS protocols. The simple web page may be deployed across hospitals that might not have FHIR and DICOMWeb server for viewing medical image and annotation.

## Conclusions

This paper introduces a novel workflow for the organization of medical images, based on FHIR APIs and associated resources in conjunction with a viewer using DICOMweb APIs to facilitate integration with existing systems. Unlike conventional imaging workflows, which require the complex HL7 V2, DICOM, and HL7 CDA specifications, the proposed scheme uses the common JSON (or XML) data and RESTful APIs of FHIR and DICOMweb. This approach facilitates implementation and management, and can be integrated with web-based HIS, the Laboratory Information System (LIS), the Pathology Information System (PIS), and other existing systems.

The proposed schemes meant to overcome barriers to system integration. With appropriate security protection, the proposed web solution could be used for the sharing of information throughout all departments inside hospital as well as across hospitals. And the secure specifications must further be addressed for cross hospital image workflows. The more detailed specifications and extensions that might be investigated further are listed as follows:

- There are three coordinate systems on the viewer, the DICOM pixel, 3D spatial coordinates and canvas coordinates, all of which could be used in image annotation. Note however that using the viewer to rotate, flip, resize, or shift the DICOM image would alter the relationship between the coordinate systems. This would be necessary to perform coordinate transformation when storing annotations on the FHIR server or displaying the annotations on the viewer. For a single image, we suggest that the DICOM pixel coordinate could be used as the reference for image annotation. However, for a series images or different series images, patient coordinate would be useful in image registry and lesion management. We are investigating the three coordinates used in various clinical domains, such as radiotherapy, surgery, etc., and would demonstrate in the future.
- It would be also worthy to note that the WADO-RS rendering might be a potential solution, which could apply to all kind of images (mammo, CT, MR, etc.) and allow the DICOMweb server to handle all the issues with 12-bit resolutions, technical factor annotations, sigmoidal lookup tables, and monochrome image interpretation, etc. [10]. Viewers usually require the ability to parse the DICOM objects. If the format of the image (e.g., row, column, bit allocation defined in the DICOM image pixel module) were known prior to retrieval, then it would be possible for the viewer to display of medical images. This would make it far easier to develop a front end system to handle imaging data by WADO-RS with rendered resources, which would directly return JPEG images to the Viewer.
- This study did not focus on the terminology or coding systems used in imaging workflows; however, we acknowledge the importance of these issues in putting together reports. For example, images for the diagnosis of breast cancer would include data pertaining to the shape, location, size, and margin of detected masses. The shape and margin must also be further defined and coded in accordance with each type of imaging modality (e.g., MRI, ultrasound, and mammography). The codes and terminology should be further investigated and carefully defined.
- The proposed FHIR imaging workflow could be used in a single facility or among multiple facilities; however, this will require further mechanisms for security and the protection of privacy. FHIR currently leverages many of the security protection mechanisms developed for the web, such as SSL and OAuth for protecting FHIR APIs [11]. However, access control to the FHIR and DICOMweb servers should also be regulated. Additionally mapping the all possible details (referring physician, accession number, visit number, admitting diagnosis, etc.) between ServiceRequest/Encounter and UPS-RS is as well a significant core items in our future work.
- We suggest using web solutions for retrieving and examining all medical images. Because of the inconvenient for image viewing in diverse systems (radiology images, biopsy video, and microscopic Whole Slide Image (WSI). It is difficult using conventional DICOM specifications to integrate an enterprise viewer. It is a great challenge for developing a viewer that can fulfill all the requirements from different clinical departments. However, as demonstrated in the paper, we can use pure web solution to present medical images and annotations on the browser. The type of zero footprint viewer do not require install any plug-in on the browser. Based on the pure web solution, different developing teams can focus on their domain and develop their own web viewing and reporting system separately. And all the results can be presented on a single browser simultaneously. It is convenient for developers to adopt FHIR specifications and create a system for clinical exam workflow. And DICOM WADO-RS is also convenient for retrieval of medical images (include video and WSI images). DICOM has addressed rendering query parameters in Part 18 8.3.5. Following the specification, we may use WADO-RS with parameters to access radiology images and WSI images. And we could also access SVG annotations from FHIR servers. The images and annotation could be rendered and presented properly on a pure web viewer. We hope to present more use cases based on standard RESTful specifications in the future.
- RESTful solution with IoT, AI, and precision medicine will bring a lot of new usages and opportunities. Medical information standards should be very important to support the potential solutions for innovative applications. However, this a huge task to define standards for the system integrating solutions in healthcare. This paper proposes an imaging integrating framework using simplified DICOMWeb and FHIR resources. The simplified specification would be easy to be understood for people who are not familiar with medical informatics standards. And developers can follow the framework described in this paper to develop the general imaging exam and report systems which may quickly be adopted into real use. There still is a lot of work for creating standard specifications for integrating AI or precision medicine system with PACS. Especially, the security specifications for the integration. It will bring lot of overhead for the security specifications to be compatible both with conventional HL7 and DICOM and with FHIR and DICOMWeb. This would constitute a very difficult to implement specifications. For the new medical imaging application, such as AI, the integration of FHIR and DICOMWeb would be the promising solution.

## Footnotes

1 All the above FHIR and DICOMweb standards are normative (Maturity Level > 5) except for DiagnosticReport (Maturity Level 3), ImagingStudy (Maturity Level 3), ServiceRequest (Maturity Level 2), and Encounter (Maturity Level 2).
